# Design and Characterization of Novel Antibody-Cytokine Fusion Proteins Based on Interleukin-21

**DOI:** 10.3390/antib11010019

**Published:** 2022-03-04

**Authors:** Cesare Di Nitto, Dario Neri, Tobias Weiss, Michael Weller, Roberto De Luca

**Affiliations:** 1 Philochem AG, 8112 Otelfingen, Switzerland; cesare.dinitto@philochem.ch (C.D.N.); dario.neri@philogen.com (D.N.); 2 Philogen SpA, Piazza la Lizza 7, 53100 Siena, Italy; 3 Department of Neurology, University Hospital Zurich, 8091 Zurich, Switzerland; tobias.weiss@usz.ch (T.W.); michael.weller@usz.ch (M.W.); 4 Clinical Neuroscience Center, University of Zurich, 8091 Zurich, Switzerland

**Keywords:** Interleukin-21, immunocytokines, protein engineering, format engineering, cytokines, immune-modulation, cross-reactivity, Solid Tumor

## Abstract

Interleukin-21 (IL21) is a pleiotropic cytokine involved in the modulation of both innate and adaptive immunity. IL21 is mainly secreted by natural killer (NK) and activated CD4+ T-cells. The biology of this cytokine can be associated to proinflammatory responses reflecting its potent stimulatory activity of NK and CD8+ T-cells. Here we describe four formats of novel IL21-based antibody–cytokine fusion proteins, targeting the extra domain A (EDA) of fibronectin and explore their potential for cancer treatment. The fusion proteins were designed, expressed, and characterized. F8 in single-chain diabody (scDb) format fused to IL21 at its C-terminus exhibited a promising profile in size exclusion chromatography (SEC) and SDS-PAGE. The lead candidate was further characterized in vitro. A cell-based activity assay on murine cytotoxic T-cells showed that human IL21, compared to murine IL21 partially cross-reacted with the murine receptor. The prototype was able to recognize EDA as demonstrated by immunofluorescence analysis on tumor sections. In an in vivo quantitative biodistribution experiment, F8(scDb)-murine IL21 did not preferentially accumulate at the site of disease after intravenous injection, suggesting that additional protein engineering would be required to improve the tumor-homing properties of IL21-based product.

## 1. Introduction

Cytokines are relatively small proteins (≈15 kDa) capable of modulating the immune system. In particular, proinflammatory cytokines have been considered for cancer therapy since they boost CD8+ cytotoxic T-cells, CD4+ helper T-cells and NK cells [1,2,3]. The systemic administration of recombinant cytokines can induce potent anti-cancer activity, leading to their marketing authorization for certain indications [4,5]. However, these immunomodulatory products may also cause side effects already at low concentrations, therefore precluding dose escalation to treatments, which would be required to meliorate the therapeutic benefit [6,7,8]. The fusion of cytokines to tumor-specific antibodies has been suggested as a strategy to increase the therapeutic efficacy of the cytokine payload, as a consequence of a specific accumulation at malignant lesions, while sparing healthy tissues [9,10]. These products (also called “immunocytokines”) are designed to reduce toxicity in patients, compared to untargeted recombinant cytokines. Many antibody-cytokine fusion proteins have been generated and tested both in preclinical mouse models of cancer and in clinical trials [11]. The validation of tumor associated antigens simplify the development of immunocytokines that specifically localize in the tumor mass as a result of a specific binding to the target antigen.

The alternatively-spliced extra domain A (EDA) and B (EDB) of fibronectin, are some of the most investigated extracellular matrix tumor-related antigens. Fully human monoclonal antibodies L19 and F8 have been isolated against EDB and EDA, respectively [7]. Both domains are conserved from mouse to man, thus easing translational activities from preclinical models to clinical studies. The tumor-targeting properties of L19 and F8 antibodies have been confirmed in mouse models of cancer and in cancer patients. Immunocytokines based on these antibody fragments are currently being evaluated in clinical trials [7].

Interleukin-21 is a pro-inflammatory cytokine secreted by activated CD4+ T and NK cells [12]. It is a member of the common cytokine receptor gamma chain family, shared by IL2, IL4, IL7, IL9 and IL15 [13].

IL21 binds its cognate receptor IL-21R which is broadly expressed on the surface of several leucocytes including CD4+ T, CD8+ cytotoxic T, NK, and myeloid cells. The IL21/IL-21R induces the activation of the JAK/STAT pathway, crucial for cell proliferation and survival of both NK and T-cells.

IL21 was proposed as an alternative to cytokines belonging to the same family such as IL2 and IL15, for cancer therapy. Compared to IL2 and IL15 which bind to a trimeric receptor (IL2β, the common gamma chain (γ_c_) and an individual alpha receptor: IL2Rα and IL15Rα) IL21 relies on the heterodimerization of IL21R with the common γ_c_.

In a comparative evaluation of IL2, IL15 and IL21, recombinant IL21 was shown to enhance and sustain CD8+ T-cell activity against tumor cells. IL21 was effective in monotherapy and cured a portion of thymoma-bearing mice whilst the activity was abrogated when the experiment was conducted in CD8+ T-cell depleted mice [14]. In another study, Shen and co-workers showed that IL21 augments the survival of CD8+ T-cells, in addition it generates a less activated but long lasting T cell phenotype, characterized by high levels of IFN, granzyme-B and perforins [15,16]. Another IL21-based fusion protein targeting epidermal growth factor receptor (EGFR) was proposed by Deng et al. In MC38-EGFR expressing tumors the fusion protein successfully localized at the tumor site. The presence of IL21 stimulated CD8+ infiltrated cells and boosted tumor-antigen specific cytotoxic activity [17].

In clinical trials, recombinant human IL21 (rIL21) was administered to metastatic melanoma (MM) or renal cell carcinoma (RCC) patients at doses ranging between 1–300 µg/kg [18]. The maximum tolerated dose (MTD) was assessed at 30 µg/kg. Davis et al. [19] studied a cohort of 24 newly diagnosed MM patients treated with rIL21, in which they observed an Objective Response Rate (ORR) of 8%. In recurrent RCC patients treated with 400 mg sorafenib and 30 µg/kg of rIL21 the ORR was 21%.

In order to increase IL21 therapeutic index, in this study, we propose four new fusion proteins consisting of the F8 antibody fused to IL21 in various formats for specific targeted delivery. The F8 antibody specifically recognizes the extra EDA domain of fibronectin, a stromal tumor-related antigen [20]. The best candidate was studied in an in vitro cell-based assay and in an in vivo quantitative biodistribution study. In the first experiment, both human and murine IL21 fusion proteins were tested to investigate species cross-reactivity.

## 2. Material and Methods

### 2.1. Cloning, Expression and Protein Purification

The fusion protein F8(scDb)-IL21 contains the antibody F8 [21] in single-chain diabody format fused to human IL21 (gene from Eurofins Genomics) at the C-terminus by a 17-amino-acid linker.

The fusion protein F8(scFv)-IL21-F8(scFv) contains the antibody F8 in single chain fragment variable format fused to human IL21 at the C-terminus by and at the N-terminus by a 10-amino-acid linker.

The fusion protein IL21-F8(scDb) contains the antibody F8 in single chain diabody format fused to human IL21 at the N-terminus by a 12-amino-acid linker.

The fusion protein F8(Db)-IL21 contains the antibody F8 in diabody format [22] fused to human IL21 at the C-terminus by a 17-amino-acid linker.

The fusion protein F8(scDb)-mIL21 contains the antibody F8 in single chain diabody format fused to murine IL21 (gene from Eurofins Genomics) at the C-terminus by a 17-amino-acid linker. Gene encoding for the F8 antibody and the gene encoding for murine IL21 were PCR amplified, PCR assembled, and cloned into the mammalian expression vector pcDNA3.1(+) (Invitrogen, Waltham, MA, USA) by a NheI/BamHI/EcoRI/NotI restriction site.

The prototypes were produced in CHO_S cells using transient gene expression (TGE) as described previously [22].

### 2.2. Protein Characterization

SDS-PAGE was achieved with 4–12% Bis-Tris gels (SurePAGE™, M00652, GenScript, Piscataway, NJ, USA) in reducing (R) and non-reducing (NR) settings. Proteins were evaluated by size-exclusion chromatography on a Superdex 200 increase 10/300 GL column on an ÄKTA FPLC (Cytiva, Marlborough, MA, USA). Affinity measurements were executed by surface plasmon resonance on a BIAcore X100 instrument (Cytiva) using an EDA coated CM5 chip. Samples were analysed in a range from 1 µM to 125 nM. Regeneration of the chip was implemented using 10 mM HCl.

### 2.3. Bioactivity Measurement

The biological activity of the fusion proteins was evaluated by their capability to induce the proliferation of murine cytotoxic T lymphocytes, CTLL2 (ATCC, TIB-214™). In 96-well plates, cells (25,000–50,000 per well) were seeded in culture medium supplemented with serial dilutions of the fusion proteins. After incubation at 37 °C for 72 h, cell proliferation was measured with Cell Titer Aqueous One Solution (Promega, Madison, WI, USA). Results were represented as the percentage of cell viability compared with unstimulated cells.

### 2.4. Cell Lines

CHO-S (CCL-61™), CTLL2 (TIB-214™) and F9 (CRL-1720™) cells were purchased from the ATCC. Cell lines were delivered between 2020 and 2021, expanded, and stored as cryopreserved aliquots in liquid nitrogen. Cells were grown according to the manufacturer’s protocol and maintained in culture for no longer than 12 passages. Authentication of the cell lines was performed by the cell bank before shipment.

### 2.5. Immunofluorescence Study

EDA expression was assessed on ice-cold acetone fixed 8-μm cryostat sections of F9 teratocarcinoma stained with F8(scDb)-mIL21 (final concentration 0.05 mg/mL) and detected with protein A-AlexaFluor488 (Invitrogen P11047). A rat anti-mouse CD31 (BD 550274) was used to detect blood vessels and revealed with donkey anti-rat ALEXA 594 (Invitrogen A21209). Omission of F8(scDb)-mIL21 was used as negative control. Cell nuclei were stained with DAPI (Invitrogen D1306). Slides were mounted with Dako fluorescent mounting medium and analysed with a Leica TIRF microscope.

### 2.6. Biodistribution Experiments

Six- to 8-week-old female 129/SvEv mice were purchased from Janvier Labs (Le Genest-Saint-Isle, France). Next, 2 × 10^7^ F9 tumor cells were injected subcutaneously in the flank. When tumors reached a volume of 100 to 200 mm^3^, F8(scDb)-mIL21 (100 µg) was labelled with ^125^I and Chloramine T, filtered on a PD10 column and inoculated into the lateral tail vein as described in [23]. Mice were euthanized 24 h after injection. Organs, blood, and tumors were weighed and radioactivity was detected using a Packard Cobra gamma counter. The immunocytokine uptake in blood, organs, and tumors was calculated and expressed as the percentage of the injected dose per gram of tissue (%ID/g ± SEM, *n* = 3). Data were adjusted for tumor growth as previously described [23]. Differences in organ uptake compared with tumor uptake were analysed using the unpaired *t*-test of Prism (GraphPad, San Diego, CA, USA) (Supplementary Table S1).

### 2.7. Ethical Statement

Animal experiments were conducted under a project permit (license number 06/2021) approved by the Veterinäramt des Kantons Zürich, Switzerland, in compliance with the Swiss Animal Protection Act (TSchG) and the Swiss Animal Protection Ordinance (TSchV).

## 3. Results

### 3.1. Generation and Format Screening of Novel IL21-Based Antibody Fusion Proteins

**Figure 1:**
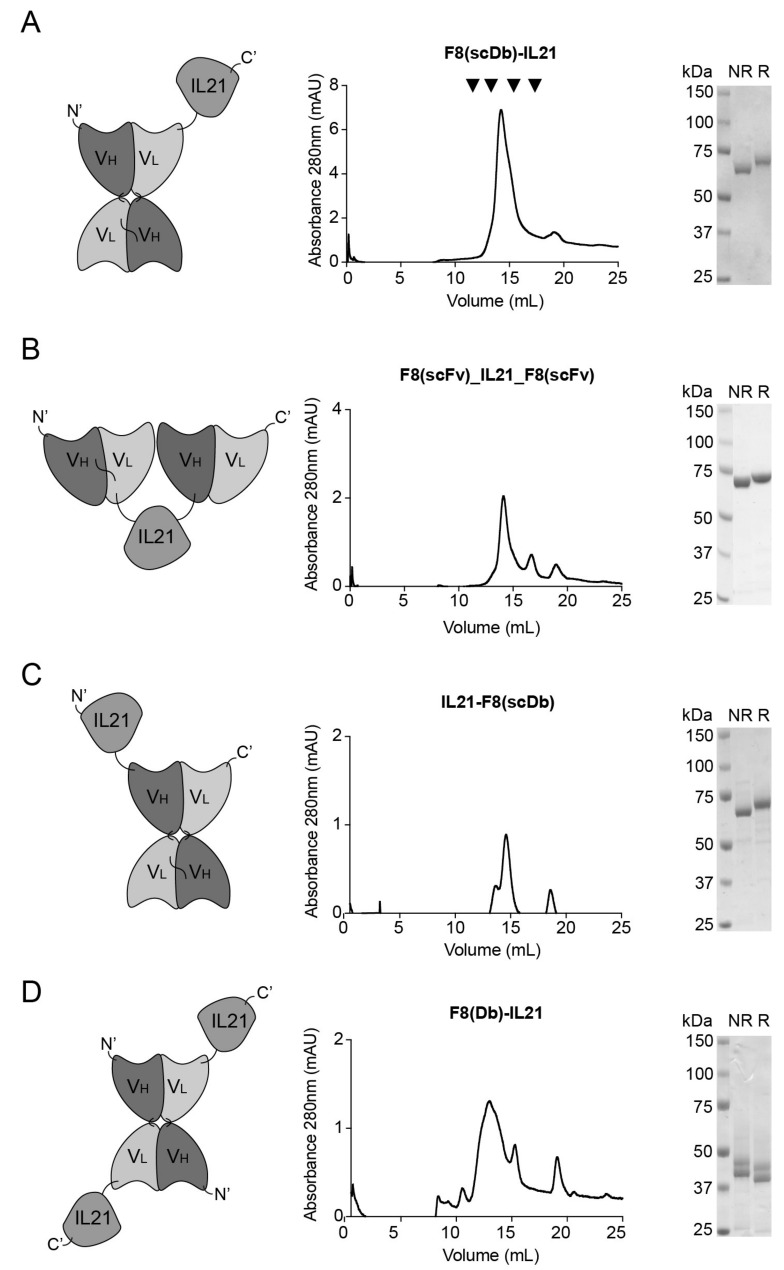
In vitro characterization of hIL21-fusion proteins. (**A**) F8(scDb)-IL21 (**B**) F8(scFv)-IL21-F8(scFv) (**C**) IL21-F8(scDb) and (**D**) F8(Db)-IL21. From left to right: schematic drawing of the antibody fusion protein; SEC profile and SDS-PAGE (NR, non-reducing condition; R, reducing condition, for the final figures gels were cut and put together). Standard references for superdex 200 increase 10/300 GL column are indicated by black arrows in A (from left to right): IgG1 146 kDa elution volume at 12 mL; Conalbumin 75 kDa elution volume at 14 mL; Ovalbumin 44 kDa elution volume at 15 mL; Carbonic anhydrase 29 kDa elution volume 16.5 mL.

Four different immunocytokines based on IL21 were generated. A first protein consisting of the F8 antibody in single chain diabody (scDb) format [24] fused at the C-terminus of human IL21 was cloned and produced in CHO-S cells (Figure 1A) with a yield of 3.9 mg/L. A second fusion protein, in which human IL21 was linked at its C- and N-terminuses by a peptide linker to the F8 antibody in single chain variable fragment (scFv) exhibited a yield of 2.9 mg/L (Figure 1B). A third fusion protein featuring of the F8 antibody in scDb format fused at the N-terminus by a peptide linker to human IL21 was cloned and produced (Figure 1C) with a yield of 1.2 mg/L. A last fusion protein in diabody (Db) format in which human IL21 was attached to the C-terminus of the F8 antibody by a peptide linker was cloned and produced with a yield of 3.1 mg/L (Figure 1D).

**Figure 2:**
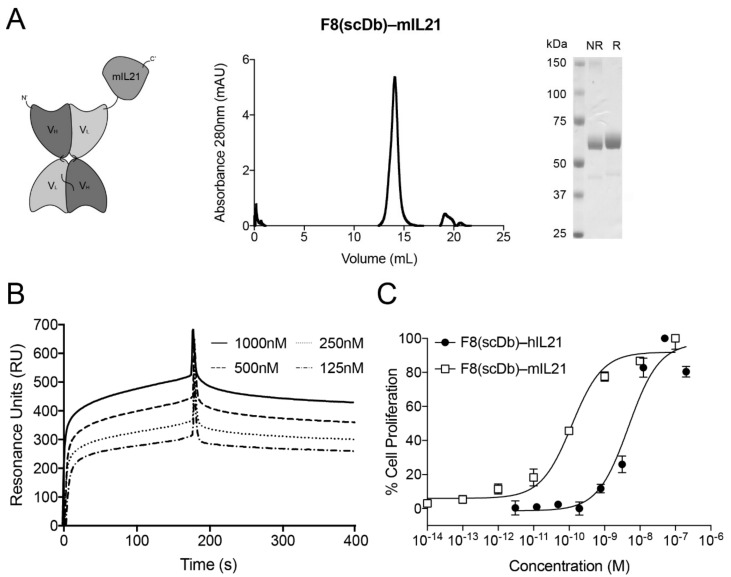
In vitro characterization and biological activity of murine and human variant in single chain diabody format with IL21 fused at the C-terminus. (**A**) F8(scDb)-mIL21; size exclusion chromatography profile; SDS-PAGE (NR, non-reducing condition; R, reducing condition). (**B**) SPR on EDA-coated chip of F8(scDb)-IL21. (**C**) Activity assay based on CTLL2 cell proliferation by exposure to F8(scDb)-mIL21 and F8(scDb)-hIL21 fusion proteins.

The best candidate F8(scDb)-IL21 was chosen based on the yields and biochemical properties (Figure 1) observed in size exclusion chromatography (SEC), SDS-PAGE, SPR and cell proliferation assays (Supplementary Figures S1 and S2). F8(scDb)-murine IL21 was used to conduct experiments in mouse models (Figure 2A). The findings are in line with previously described fusion protein based on the scDb format [22,25,26].

### 3.2. Biochemical Properties and Activity of F8(scDb)-IL21

A surface plasmon resonance analysis confirmed that the F8 antibody retains binding to EDA when fused to IL21 (Figure 2B). An in vitro lymphocyte proliferation analysis on murine CTLL2 cells indicated that human IL21 was about 10-fold less active than murine IL21 (i.e., EC50 of 4.7 nM for human IL21 and 0.1 nM for murine IL21).

### 3.3. In Vitro Immunofluorescence Analysis

**Figure 3:**
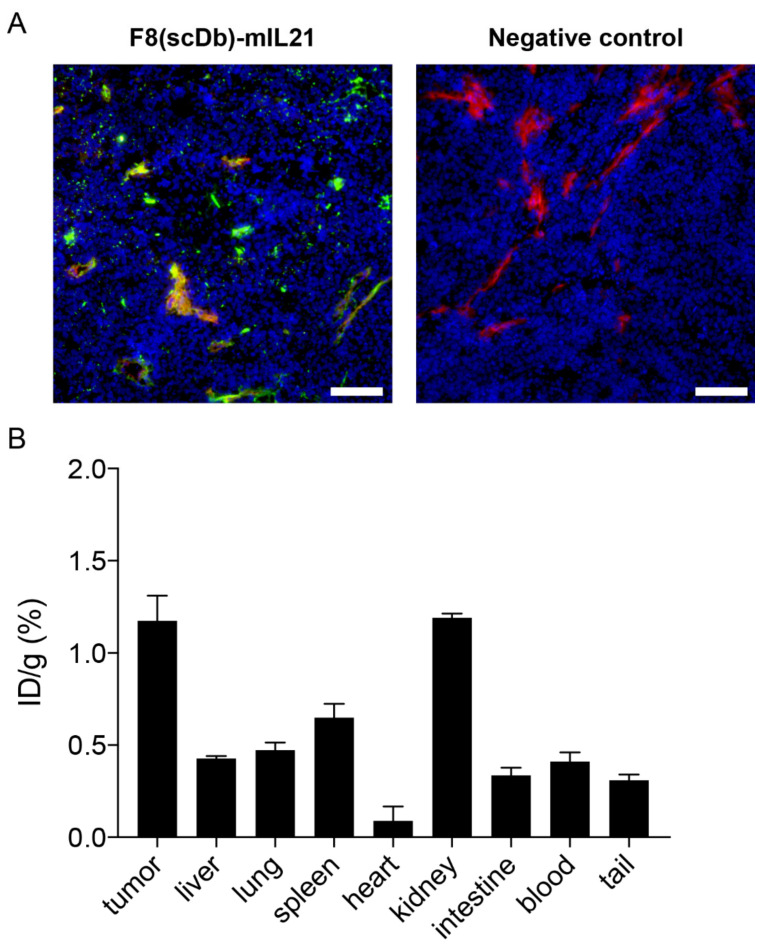
Tumor targeting properties of F8(scDb)-mIL21. (**A**) Microscopic fluorescence evaluation of EDA levels on F9 tumor sections detected with F8(scDb)-mIL21 (green, ProteinA AlexaFluor488). Blood vessels are stained with anti-CD31 (red, AlexaFluor594). Cell nuclei are stained with DAPI (blue). Omission of F8(scDb)-mIL21 was included as negative control, 20× magnification, scale bars = 100 μm. (**B**) Quantitative biodistribution analysis. of F8(scDb)-mIL21 was labelled with ^125^I and injected into the lateral tail vein of F9 tumor-bearing 129/sv mice. After 1 day, mice were euthanized, organs, blood and tumors were removed, weighed, and radioactivity detected. Plots are expressed as the percentage of the injected dose per gram tissue (%ID/g SEM). Data were adjusted for tumor growth.

An in vitro immunofluorescence analysis revealed that F8(scDb)-mIL21 strongly stained the vasculature of F9 teratocarcinomas (Figure 3A). This result confirmed expression of fibronectin EDA in the F9 tumor model [27] and effective recognition of the IL21-fusion protein to its cognate antigen.

### 3.4. In Vivo Tumor Targeting Performance

In vivo tumor targeting properties of radio-labelled F8(scDb)-mIL21 were evaluated by quantitative biodistribution analysis in immunocompetent mice, grafted with subcutaneous F9 teratocarcinomas. The fusion showed inadequate targeting performance 1 day after intravenous administration and a tumor:blood ratio of 2.8 (Figure 3B and Supplementary Table S1).

## 4. Discussion and Conclusions

In this study, we compared different immunocytokine formats based on the F8 antibody fused to IL21. Among these fusion proteins, F8(scDb)-IL21 exhibited favorable biochemical properties suitable for further in vitro and in vivo assessments. Both human and murine IL21-fusions were produced to investigate species cross-reactivity. Cross-reactivity among species is an important, although not extensively addressed, feature of many biologically active payloads [28]. In the pharmaceutical industry, cross-reactivity of a specific product can often lead to an accelerated process when bringing a novel prototype to the clinical stage. For instance, in the case of immunocytokines, cross-reactivity allows usage of the same product both for pre-clinical and clinical investigations. In this work, we have shown in a cell proliferation assay that the human payload only partially boosts murine T cells. For this reason, it is more suitable to conduct pre-clinical studies with the murine IL21 surrogate.

Regardless of the in vitro immunofluorescence analysis showing binding of the prototype to its target antigen, the in vivo quantitative biodistribution study revealed insufficient accumulation of F8(scDb)-mIL21 to neoplastic lesions. A possible explanation may be attributed to the radioiodination process. The methodology could, in theory, prevent an antibody from binding to its cognate antigen. Chloramine T was used as an oxidative agent to perform the radiolabeling reaction where ^125^I is substituted for reactive hydrogen sites in the target molecules [29,30]. Chloramine-T could indeed act not only as an oxidizing agent for the sodium iodide, but also chemically modify and damage the antibody-cytokine fusion [31,32]. In the past, we reported a potential competition among the antibody-mediated tumor targeting and the capture of immunocytokines by the cytokine receptor expressed on healthy cells [33,34], it is therefore possible that a receptor trapping for IL21 might explain the insufficient accumulation to the tumor. The insufficient accumulation to neoplastic lesions, confirm that the generation of immunocytokines with suitable tumor-homing properties and their detection is not straightforward since multiple factors can play a negative role.

In conclusion, we have generated a novel IL21-based antibody fusion protein featuring promising biochemical and immunostimulatory properties, but with suboptimal in vivo targeting abilities. For this reason, additional protein engineering studies (e.g., screening of different linker length and amino acid composition between the fusion protein domains [35], design of additional antibody–cytokine fusion formats [36] and/or muteins of IL21 with reduced binding to IL21R(15)) could be explored to enhance the tumor-homing properties, crucial for the development of IL21-targeted biopharmaceuticals.

## Data Availability

The data presented in this study are available on request from the corresponding author.

## Supplementary Materials

The following supporting information can be downloaded at: https://www.mdpi.com/article/10.3390/antib11010019/s1, Figure S1: Surface Plasmon Resonance on EDA-coated sensor chip of (A) F8(scDb)-IL21; (B) IL21-F8(scDb); (C) F8(scFv)-IL21-F8(scFv) and (D) F8(Db)-IL21; Figure S2: Activity assay based on CTLL2 cell proliferation by exposure to (A) F8(scDb)-IL21; (B) IL21-F8(scDb); (C) F8(scFv)-IL21-F8(scFv) and (D) F8(Db)-IL21; Table S1: Statistical analysis of biodistribution experiment. Differences in organ uptake compared with tumor uptake were analysed using the unpaired *t*-test of Prism.
