# Supplementary material for: Design and Characterization of Novel Antibody-Cytokine Fusion Proteins Based on Interleukin-21
Source: Antibodies (Basel). 2022 Mar 4;11(1):19. doi: 10.3390/antib11010019 (PMC8944420; doi:10.3390/antib11010019)
Supplement: Supplementary file 1 [file antibodies-11-00019-s001.zip › antibodies-1581592-supplementary.pdf]

Supplementary Information

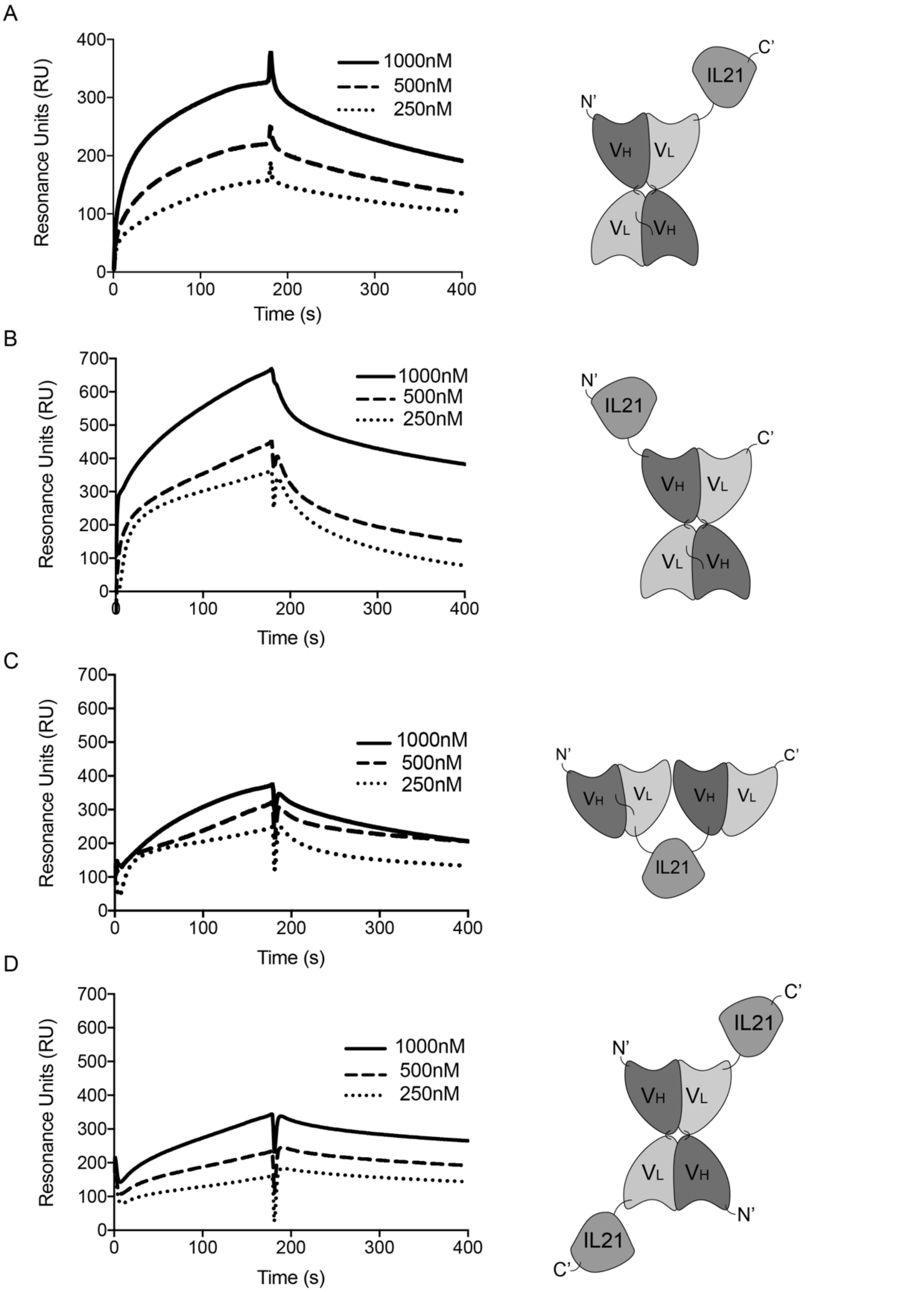

Figure S1. Surface Plasmon Resonance on EDA-coated sensor chip of (A) F8(scDb)-IL21; (B) IL21-F8(scDb); (C) F8(scFv)-IL21-F8(scFv) and (D) F8(Db)-IL21.

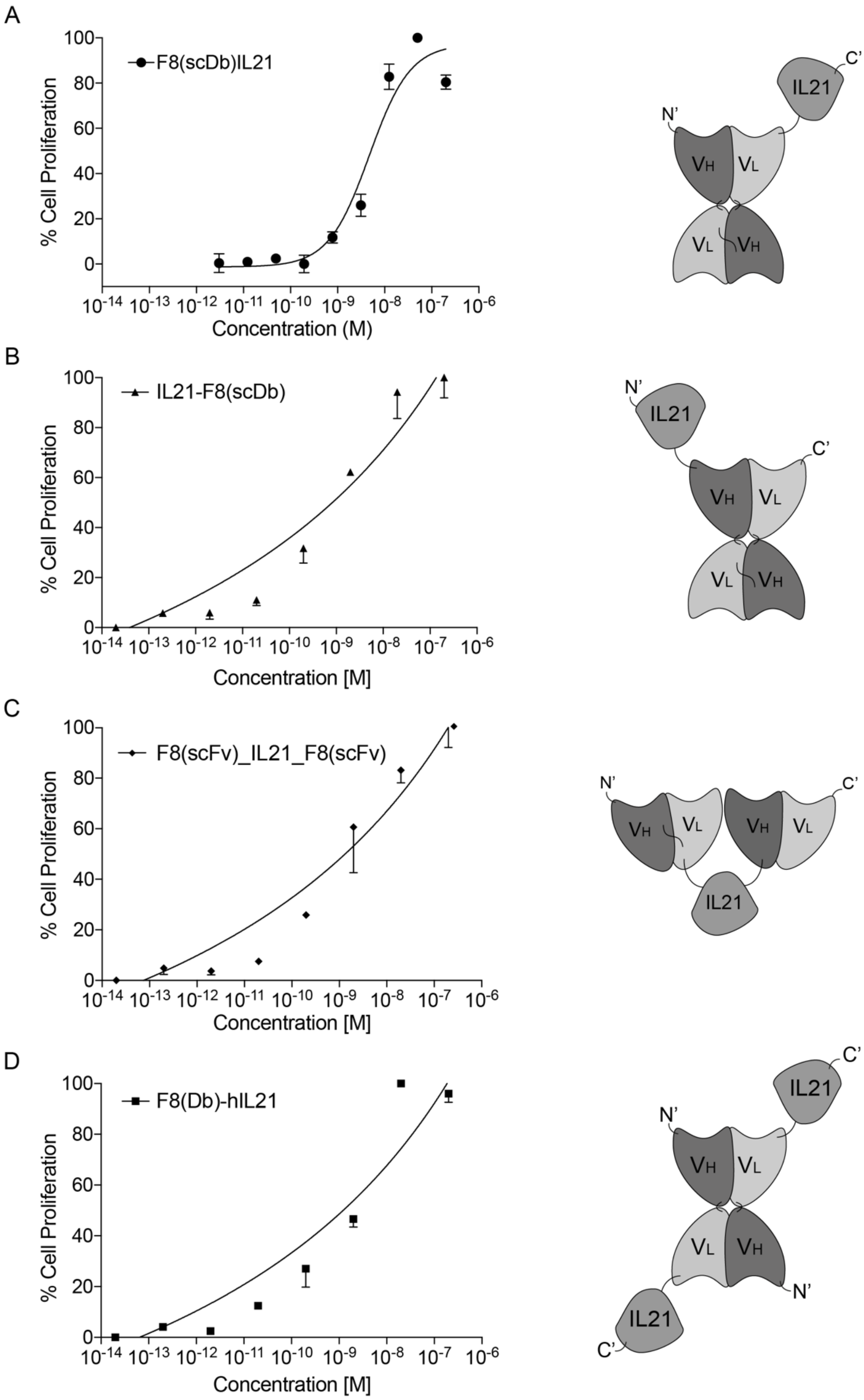

Figure S2. Activity assay based on CTLL2 cell proliferation by exposure to (A) F8(scDb)-IL21; (B) IL21-F8(scDb); (C) F8(scFv)-IL21-F8(scFv) and (D) F8(Db)-IL21.

Table S1. Statistical analysis of biodistribution experiment. Differences in organ uptake compared with tumor uptake were analysed using the unpaired t-test of Prism (GraphPad).

Tumor vs Liver	$p = 0.0158$
Tumor vs Lung	$p = 0.0225$
Tumor vs Spleen	$p = 0.0534$
Tumor vs Heart	$p = 0.0060$
Tumor vs Kidney	$p = 0.9930$
Tumor vs Intestine	$p = 0.0132$
Tumor vs Blood	$p = 0.0159$
Tumor vs Tail	$p = 0.0101$
